# Antibacterial applications of biologically synthesized *Pichia pastoris* silver nanoparticles

**DOI:** 10.1016/j.heliyon.2024.e25664

**Published:** 2024-02-06

**Authors:** Pragati Rajendra More, Surbhi Shinde, Zhejiang Cao, Jian Zhang, Santosh Pandit, Anna De Filippis, Ivan Mijakovic, Massimiliano Galdiero

**Affiliations:** a Department of Experimental Medicine, Section of Microbiology and Clinical Microbiology, University of Campania “L. Vanvitelli, ” Via De Crecchio, 7, 80138, Naples, Italy; b Systems and Synthetic Biology Division, Department of Life Sciences, Chalmers University of Technology, 41296, Gothenburg, Sweden; c Novo Nordisk Foundation Center for Bio Sustainability, Technical University of Denmark, 2800 Kongens Lyngby, Denmark

**Keywords:** *Pichia pastoris*, Silver nanoparticles, Antimicrobial, Multidrug resistant bacteria

## Abstract

**Objectives:**

This article highlights the biological synthesis of silver nanoparticles (AgNPs) with their characteristic analysis, and it focuses on the application of synthesized NPs against multidrug resistance (MDR) bacteria. A cytotoxicity study was performed to assess the biocompatibility.

**Methods:**

Silver nanoparticle (AgNPs) formation was confirmed by different characterization methods such as UV–Vis spectrophotometer, Dynamic light scattering (DLS)- Zeta, Fourier transform infrared (FTIR), and Transmission electron microscope (TEM). The antimicrobial activity of the AgNPs was checked against various bacterial strains of *Staphylococcus aureus (S. aureus), Escherichia coli (E. coli), Enterococcus faecalis (E. faecalis), and Klebsiella pneumonia (K. pneumonia)* by disc diffusion, minimum inhibition concentration test (MIC), and kinetic studies. The cytotoxicity of NPs against the Vero cell line was studied by cytotoxic assay.

**Results:**

The primary analysis of the formation of nanoparticles (NPs) was made by UV–Vis spectrophotometric analysis at 400 nm. At the same time, the efficient capping checked by FTIR shows the presence of a functional group at different wavelengths 3284, 1641,1573,1388,1288, and 1068 cm^−1^. At the same time, the transmission electron microscopic analysis (TEM) and DLS show that the shape and size of the synthesized NPs possess an average size of around ∼10–30 nm with spherical morphology. Further, the zeta potential confirmed the stability of the NPs. While the yield of NPs formation from silver salt was determined by an online yield calculator with the EDX analysis results. Synthesized NPs showed bactericidal effects against all the selected MDR pathogens with nontoxic effects against mammalian cells.

**Conclusion:**

Our findings indicate the remarkable antimicrobial activity of the biologically synthesized AgNPs, which can be an antimicrobial agent against multi-drug-resistant bacteria.

## 1 Introduction

In recent years, the number of microorganisms that have acquired resistance to commercially available antibiotics has increased dramatically due to the overuse of known antibiotics. This ultimately leads to the resistance of various bacterial strains to drugs due to the lack of effective antibiotics [1]. Multidrug resistance (MDR) is bacterial resistance causing a steady increase in mortality and morbidity worldwide. The clinical challenges lead to developing new efficient alternative treatments for MDR bacterial pathogens. In recent years, bionanotechnology has been used as an alternative treatment method in the medical field. The unique properties of nanoparticles (NPs), which can penetrate the cell membrane of the pathogenic organism and disrupt key pathways, make them an effective alternative to commercially available antibiotics. These NPs also act as a synergistic approach combined with other antibiotics and can curb the spread of global MDR crises worldwide [2]. The medicinal applications of silver have been known since ancient times, and in recent years, the use of this ancient knowledge to synthesize silver nanoparticles has increased [[3], [4], [5]]. This is due to their exceptional physical and chemical properties. The various applications of these AgNPs in catalysts [6], biopharmaceuticals [7], wastewater treatment [8], additives in fertilizers [9], and biomedicine [10]. The AgNPs of 1–100 nm have numerous physical, chemical, and biological properties depending on the size, shape, structure, and composition [6]. The top-down or bottom-up protocol for synthesizing NPs by the conventional physical or chemical method [11,12]. However, these methods are energy and cost intensive. Moreover, the chemicals used for synthesis are highly toxic. However, the biological synthesis

of AgNPs using natural extracts or microbial fermentation broth in the presence of silver ions has several advantages over chemical synthesis [[13], [14], [15]]. The biologically synthesized AgNPs have common physical properties and unique biological activities such as antimicrobial activity [16]. Due to high throughput and lower cost, microbial synthesis of AgNPs is the most popular. It includes microorganisms, plant leaves, fungi [17], mushroom [18,19] yeast [20], and actinomycetes [21] as reducing agents.

This study used a yeast extract of *Pichia pastoris* GS115 as a reducing agent. The yeast strain is widely known for its ability to give a high yield of recombinant protein. It exhibits inherent reducing properties that enhance the conversion of silver ions (Ag+) into AgNPs. Leveraging the intracellular reducing equivalents, such as NADH or NADPH, *Pichia pastoris* GS115 efficiently facilitates the synthesis of AgNPs within its cellular milieu [22]. The unique synthesis approach utilizing this yeast strain offers several advantages, including large-scale production potential, controlled synthesis conditions, and natural reducing agents within the yeast cells. These factors contribute to the enhanced stability, reproducibility, and scalability of the synthesized AgNPs [23].

The biological synthesis of silver nanoparticles from *Picha pastoris* and their antimicrobial applications is the aim of this study. The synthesized NPs were characterized, and their antimicrobial potential was verified. They were tested against various MDR bacterial pathogens.

## 2 Material and method

### 2.1 Material

Silver nitrate (AgNO_3_,≥99 %), Mueller Hinton agar, Mueller Hinton broth, 3-(4, 5-dimethylthiazol-2-yl)-2, 5-diphenyltetrazolium bromide (MTT), Dulbecco's Modified Eagle's Medium (DMEM) supplemented with 10 % fetal bovine serum (FBS), 1 % penicillin-streptomycin solution. All solutions were prepared in distilled water and purchased from American Type Culture Collection (ATCC Manassas, VA, USA), and the *Pichia pastoris* strain was ordered from Invitrogen GS115, Pichia pastoris Yeast Strain (Catalog number: C18100).

### 2.2 Tested microorganisms

Multidrug-resistant (MDR) clinical isolates were collected at the Unit of Virology and Microbiology of University Hospital “Luigi Vanvitelli,” Naples, Italy. Bacterial identification was carried out via MALDI-TOF MS (Bruker Daltonics, Bremen, Germany) and BD Phoenix system (Becton Dickinson, USA). Antibiotic resistance patterns were evaluated. The MDR bacterial strain resistance patterns are reported in Table 1.

**Table 1:** Antibiotic resistance profiles of MDR strains were used in the study.

Antibiotic Resistance Profile of the Clinical Isolated Bacteria		
*S. aureus* MDR		
Fusidic acid	0.5	S
Daptomycin	0.25	S
Erythromycin	4	R
Fosfomycin	64	R
Gentamicin	8	R
Linezolid	2	S
Levofloxacin	8	R
Oxacillin	2	R
Teicoplanin	0.5	S
Tetracycline	1	S
Tigecycline	0.12	S
Trimethoprim/sulfamethoxazole	20	S
Vancomycin	0.5	S
Penicillin	0.25	R
Rifampicin	2	R
*E. faecalis* MDR		
Ampicillin	8	S
Gentamicin/syn	500	R
Imipenem	8	R
Linezolid	2	S
Teicoplanin	0.5	S
Tigecycline	0.25	R
Vancomycin	2	S
*E. coli* MDR		
Amikacin	16	
Amoxicillin/clavulanate	32/2	R
Ampicillin	8	R
Cefepime	8	R
Cefotaxime	4	R
Ceftazidime	8	R
Cefuroxime	8	R
Ciprofloxacin	1	R
Ertapenem	0.25	R
Fosfomycin	16	R
Gentamicin	4	R
Imipenem	0.25	R
Levofloxacin	2	R
Meropenem	0.125	R
Piperacillin	16	R
Piperacillin/tazobactam	16/4	R
Tobramycin	4	R
Trimethoprim/sulfamethoxazole	1/19	R
Tigecycline	1	R
*K. pneumoniae* MDR		
Ciprofloxacin	4	R
Fosfomycin	64	S
Ampicillin	1	S
Gentamicin	320	R
Trimethoprim/sulfamethoxazole	64	R
Amikacin	32	R
Amoxicillin/clavulanate	8	R
Cefepime	64	R
Cefotaxime	64	R
Cefotaxime	64	R
Ertapenem	8	R
Imipenem	16	R
Meropenem	16	R
Piperacillin/tazobactam	128	R
Colistin	0.5	S

### 2.3 Preparation of yeast extract

**Fig. 1:**
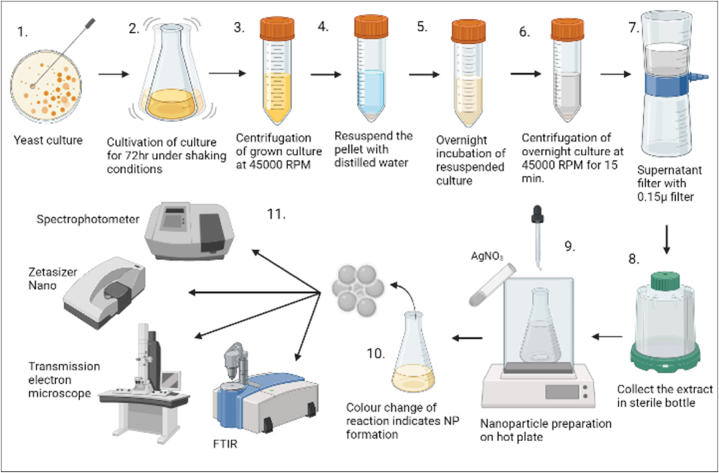
Graphical representation of extract to nanoparticle synthesis. (1) Loopfull of grown yeast culture collected and inoculated in 500 ml flask. (2) The flask was kept under constant shaking conditions for 72 h (3) Culture in the flask was transferred to a flacon and centrifuged at 45000 RPM (4.) Resuspended the pellet with distilled water (5.) Resuspended culture tubes were stored at 37 °C overnight. (6.) Overnight intubated tube was centrifuged at 45000RPM for 15 min (7) The collected supernatant was filtered through a 0.15μ filter (8). The filtrate is used as the extract and stored in a sterile bottle for further use. (9) 100 ml of extract is added in a conical flask while the flask is kept on a hot plate at 60 °C, to which drop-wise silver nitrate was added. The reaction was conducted for 48 h at 60 °C in the dark under constant shaking conditions. (10) Reaction color changes from colorless to dark yellow indicate the NPs formation. (11) Further, the NPs formation was confirmed with the different characterization methods such as UVis-spectrophotometer, Zetasizer (for DLS and Zeta potential), TEM, and FTIR. (For interpretation of the references to color in this figure legend, the reader is referred to the Web version of this article.)

*P. pastoris* strain was grown on YPD broth in a 100 mL Erlenmeyer flask containing 50 mL YPD broth, and a loop full of yeast culture was inoculated. The flask was further incubated for 72 h at 37 °C with constant shaking at 600 rpm. After 72 h of incubation, media color change was observed, and OD was checked at 600 nm. The OD was found around 10^−4^-10^−5^; then, the yeast culture was transferred in a 15 mL Falcon tube, which was centrifuged at 45000 rpm at 37 °C for 10 min. After the centrifugation supernatant was discarded, the pellet was resuspended with sterile distilled water. The container with resuspended water was kept for overnight incubation. The next day, a resuspended solution was centrifuged at 45000 rpm for 15 min, the supernatant was filtered with a 0.15μ filter, and the filtrate was collected in a sterile bottle. The stored filtrate was used as a reducing agent to synthesize AgNPs. The entire yeast extraction process was carried out under sterile conditions (Fig. 1).

### 2.4 Biological synthesis of AgNPs

The biological synthesis of AgNPs was carried out in the presence of 10 mL yeast extract in an Erlenmeyer flask. The flask was kept on an orbital shaker under constant shaking at 600 RPM at 60 °C. Then, 90 ml dropwise, 1 mM (1 mM) of an aqueous silver nitrate solution was added. On the orbital shaker, the reaction was kept at 60 °C for 48 h in the dark with constant shaking. Gradually, the color of the reaction changed from colorless to dark yellow. After 48 h, this reaction mixture was centrifuged and washed several times with water while the centrifugation helped remove the untreated salts and extract to perform the dry pellet. This pellet was kept at 40 °C to acquire the NPs powdered form.

### 2.5 Characterization

#### 2.5.1 UV–vis spectrophotometer

The surfaces of the NPs have unique optical properties due to their size, shape, concentration, and refractive index. To analyze these properties, UV–Vis spectrophotometer analysis was performed. In this study, a VWR PV4 spectrophotometer was us to investigate the synthesized AgNPs.

#### 2.5.2 DLS and zeta potential

The average particle size and charge on the surface of the NPs were analyzed using DLS and zeta potential. The analysis was performed using the Zetasizer Nano instrument (ZEN3600).

#### 2.5.3 FTIR

Attenuated total reflectance is Fourier-transform infrared (ATR-FTIR) was recorded on Bruker's Alpha spectrometer using diamond crystal as the refractive element. Spectra were acquired from the average of 256 scans at a resolution of 4 cm^−1^.

#### 2.5.4 TEM

The synthesized NPs morphology and size were confirmed using TEM by placing a drop of the dispersed solution on a carbon-coated copper grid and then drying it before analysis. The analysis was performed using FEI Tecnai T20 at 200 kV TEM/STEM with a LaB6 filament. (TEM) integrated with Energy Dispersive X-ray (EDX) Analyzer. A TESCAN Vega TS 5136LM, typically at 20 kV at a working distance of 20 mm, and a Tecani F20 TEM at 200 kV voltage were used. While determining the percent of NPs formation in the solution, an online tool named Instanano was used to determine the concentration of the solution [24].

### 2.6 Antimicrobial activity

#### 2.6.1 Disc diffusion

The antimicrobial potential of biologically synthesized AgNPs was tested against various MDR pathogens. Kirby-Bauer's disc diffusion method was used to evaluate the susceptibility of the NPs. This study was analyzed using different MDR bacterial strains, namely *E. coli*, *E. faecalis*, *K. pneumonia*, and *S. aureus*. Briefly, fresh colonies were used to prepare an inoculum at 0.5 McFarland turbidity. The bacterial suspension was homogeneously plated on Muller Hinton agar plates (Oxoid, Basingstoke, Hampshire, MA, USA). The sterile blank disc was used to load the NPs sample. This paper disc was placed in the center of the Petri plate, and 10 μL of AgNPs was added. At the same time, a Vancomycin (Thermo Fisher Scientific, Waltham, MA, USA) was used as a positive control against Gram-positive bacteria, and an Ampicillin was used against Gram-negative bacteria (Thermo Fisher Scientific, Waltham, MA, USA) as a positive control. Further, the plates were kept at 37 °C for 24 h of incubation. After 24 h of incubation, a diffusion zone was observed around the paper disc.

#### 2.6.2 Minimum inhibitory concentration test

The microdilution method calculates the minimum inhibitory concentration (**MIC)** values of the synthesized NPs. In 96-well plates, the NPs were serially diluted in the 0.78–100 μg/m range. The same dilution was used in the presence of ampicillin and vancomycin as positive controls for Gram-positive and Gram-negative bacterial strains. In addition, untreated bacteria were used as a negative control. A bacterial suspension of approximately 1 × 10^−6^ CFU/mL was adjusted, and 50 μL of this inoculum was added to each well to achieve a 5 × 10^−5^ CFU/well concentration. This plate is incubated at 37 °C for 24 h.

#### 2.6.3 Time-killing assay

To clarify the kinetics of NPs against bacteria, the time-killing test was performed per the American Society for Testing and Materials International (ASTM) standard guidelines and following the protocol described by Loo et al. [25] In the time-killing assay that was performed as described by Zainin et al. in an MHB medium, the bacterial OD value used in this study is 10^−6^ CFU/mL. To obtain the final concentration of 2 MIC, MIC, ½ MIC for each bacterial strain in the final total volume of 1 mL. At 37 °C with 150 RPM speed, samples were incubated on MHA plates (100 μL). The culture was spread at different time intervals of 0,1,2,4 and 24 h. This experiment was carried out in triplicate, and the colony number on MHA plates was calculated in CFU/mL after incubation at 37 °C for 24 h. The viable CFU was plotted against treatment time (time-kill curve). It is compared with the control positive and controls negative angles to understand whether the effect is bactericidal or bacteriostatic.

### 2.7 Cytotoxicity assay

To investigate the cytotoxicity of AgNPs, an MTT reduction assay was performed on the African green monkey cell line, namely Vero. In a tissue culture flask, cells were grown in DMEM with 10 % FBS (inactivated) and 1.0 % streptomycin-penicillin. After reaching 70–80 % confluence, cells were harvested using 2 % trypsin in DMEM. 5 × 10^3^/well cells density were plated in 96-well plates (Day 0). Further, the plate was incubated for 24 h at 37 °C with 5.0 % CO_2_. AgNPs treatment was given after 24 h incubation, at different concentrations (Day-1), and then this plate was incubated for 24 h. The next day (Day 2), after adding MTT solution to each well, plates were incubated for 4 h. The viable cells reduce the MTT to formazan. After 4 h, this formazan dissolved by adding DMSO. At 570 nm, the absorbance cytotoxic effect was measured with a plate reader [26].

#### 2.7.1 Statistical analysis

The data are expressed as mean ± standard deviation (SD) of three independent experiments. By using the Prism 9.0 software (Graph Pad, San Diego, CA, USA), post-hoc Dunnett's and One-way analysis of variance (ANOVA) tests were performed. Significant differences were considered at a p-value <0.05.

## 3 Results

### 3.1 Synthesis and characterization of AgNPs

**Fig. 2:**
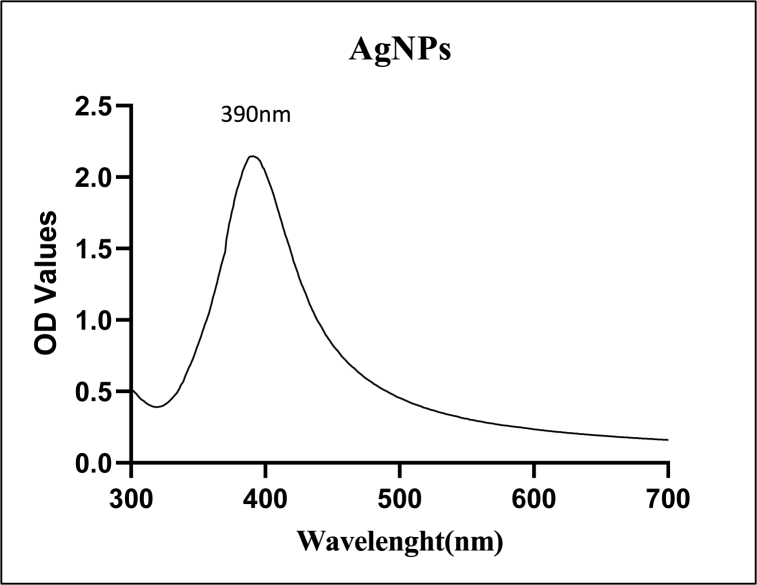
UV–Vis spectrophotometer wavelength of synthesized AgNPs was 390 nm.

AgNPs synthesis by the biological synthesis method was performed in the presence of yeast extract and 1 mM silver nitrate in equal volumes. The addition of the yeast extract changes the color of the reaction from colorless to dark yellow. The color change of a solution is considered the end point of the reaction; the corresponding reaction was continued for 120 min with constant shaking and at a temperature of 60 °C. At the same time, the color change of the reaction was confirmed by UV–Vis spectrophotometric analysis. The average peak was between 300 and 700 nm; in this study, a sharp peak was observed at 390 nm (Fig. 2.).

### 3.2 Nanoparticles stability

In the biological synthesis of AgNPs from *Pichia pastoris* extract, Ag^+^ ions were reduced by yeast extract as a source of a natural reducing agent. The yeast extract's vitamins, amino acids, and carbohydrates act as reducing agents. The biomolecules help stabilize the synthesized AgNPs. The affinity of the NPs to bacterial membranes is due to the surface coating, which may also enhance the interaction with the peptidoglycan of the bacterial cell wall. That leads to a change in the configuration of the peptidoglycan, eventually to apoptotic damage of the bacterial cell and, ultimately, death of bacteria.

As the yeast extract (*Pichia pastoris*) was added to the AgNO_3_ solution, the reaction color changed from colorless to dark yellow. After 120 min of reaction, due to the reduction of Ag ions from Ag ^+^ to Ag^0^. The color change of the response indicates the endpoint of the reaction. Different characterization instruments further analyze this reaction. The UV–Vis spectroscopy shows the structural characteristics of synthesized NPs. In this study, a strong peak was observed at 390 nm. Fig. 2.

**Fig. 3:**
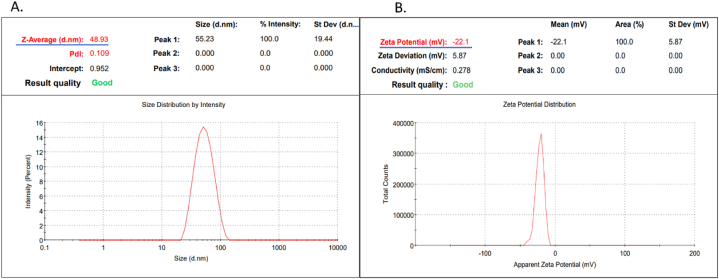
Analysis of NPs size and charge of NPs (A.) Indicates the size of NPs 48 nm while the polydisperse index is 0.154. (B) The Zeta potential of synthesized AgNPs is −22.1.

Further, to understand the size, surface charge, and stability of AgNPs DLS, zeta potential and FTIR analysis were performed. The average size of particles was in the range of 10–30 nm. While the DLS analysis shows a sharp peak at 48.93 nm Fig. 3A. At the same time, the zeta potential shows a single sharp peak at −22.1 Fig. 3 B. Which is also having low polydispersity index (PDI) values indicating the highest stability of synthesized NPs.

**Fig. 4:**
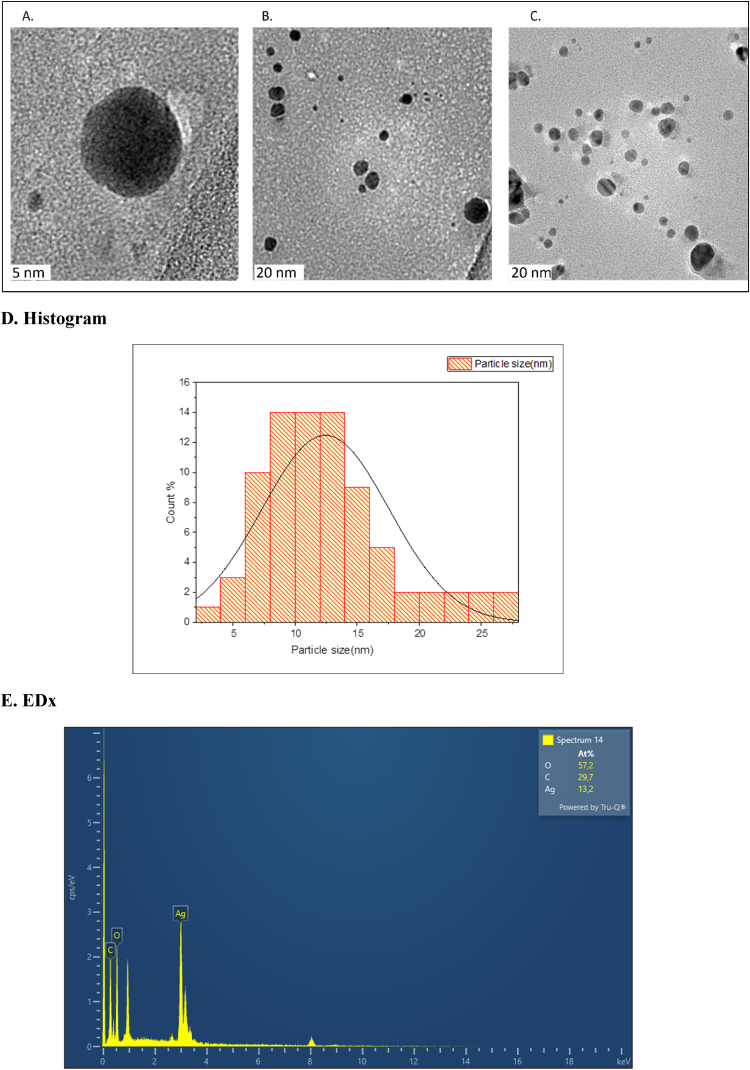

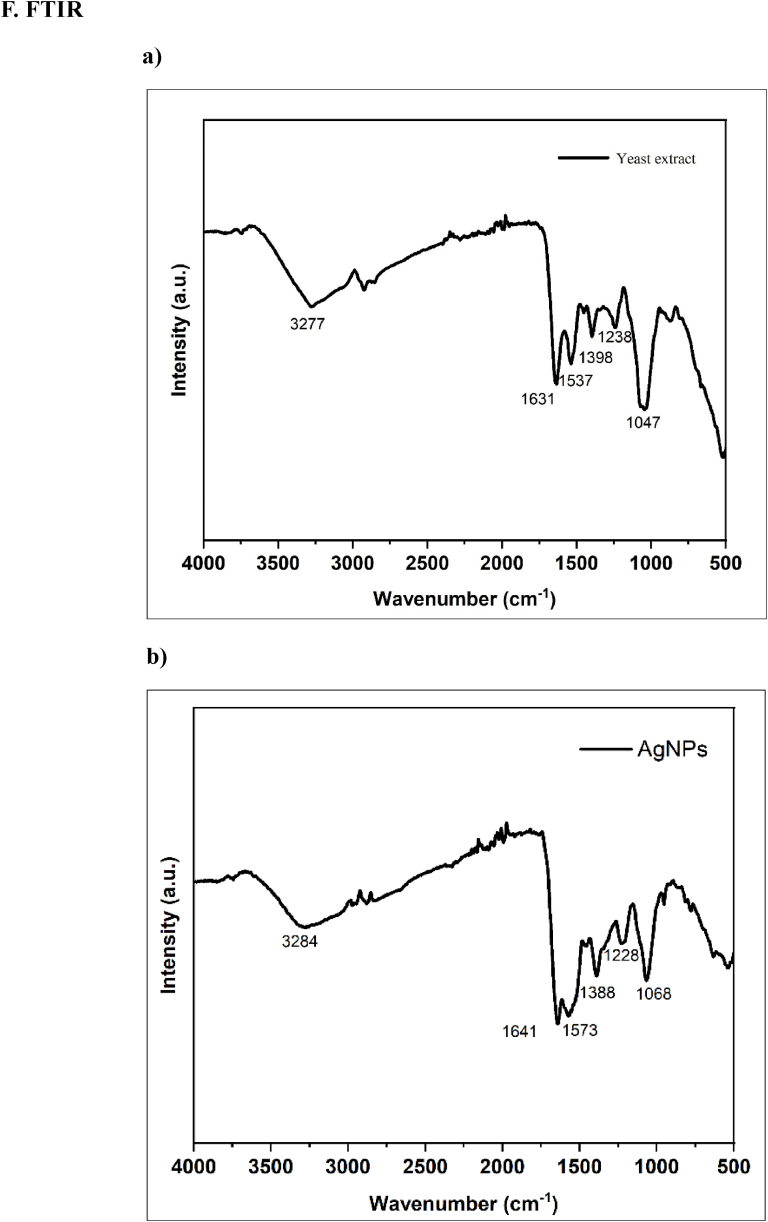
(A, B, and C) Indicates the TEM images of synthesized AgNPs, and the average size of NPs is 5–20 nm with a spherical shape. (D) Size distribution histogram explains the average particle size in between 10 and 30 nm (E) EDx analysis spectrum showing a strong peak at 3kev (F) Graphical representation of the FTIR analysis with the different functional groups figure (a). Indicate the functional group present in the yeast extract. (b.) Indicate biologically synthesized AgNPs while figure.

FTIR analysis is an essential process to analyze the involvement of functional groups in synthesizing NPs. The FTIR spectra recorded the difference in wavelengths or change in intensity shift, which gives an idea regarding which functional groups are present in the binding mechanisms of NPs synthesis. In this study, we recorded the FTIR spectra for the yeast extract and the AgNPs. A comparative analysis of the FTIR spectra indicated in (Fig. 4F) a simplified spectrum, suggesting a selective capping of *P. pastoris* extract components covering the metal NPs. FTIR analysis also provides insight into the encapsulation and stability of NPs because the reduction of metal ions is due to secondary metabolites in the biological extract. In this study, we observed different peaks at a wavelength of 3277 cm^−1^, 1631 cm^−1^, 1537 cm^−1^, 1398 cm^−1^, 1238 cm^−1^, and 1047 cm^−1^ in extract, and in AgNPs shows 3284 cm^−1^, 1641 cm^−1^,1573 cm^−1^, 1388 cm^−1^, 1288 cm^−1^, and 1068 cm^−1^. The functional group followed at 3277 cm^−1^ -3284 cm^−1^ can belong to the N–H stretching of a secondary amine and weak broad OH stretching alcohol (H2O) [27]. The 1631 cm^−1^-1641 cm^−1^ and 1398 cm^−1^-1573 cm^−1^ peaks indicate the C=O stretching vibration and N–H stretching bending in their amide linkages. In addition, the 1641 and 1631 cm^−1^ peaks may as well be from the C=C bond of aromatic compounds. Also, the 1398 cm^−1^-1388 cm^−1^ peak can be representative of the C–H bending vibrations of carbohydrates. Peaks at 1288 cm^−1^ and 1238 cm^−1^ and 1068 cm^−1^ and 1047 cm-1 are contributed from C–O and C–O–C stretching, respectively [28,29]. Fig. 4 TEM observed the shape and size of the synthesized NPs. The resulting AgNPs possess a spherical shape, with the average size of NPs in the 10–30 nm range. Fig. 4. The elemental constituent and relative abundance of the biosynthesized AgNPs were obtained from EDX, as presented in (Fig. 4. E). The EDX spectrum (Fig. 4. E) reveals the purity and the complete chemical composition of AgNPs. The reduced AgNPs were subjected to EDX analysis with an optical composition of elements showing atomic percentage, such as oxygen(O)57.2 %, carbon(C) 29.7 %, and silver (Ag)13.2 %. The other elements served as capping organic agents bound to the surface of AgNPs. While the TEM analysis gives insight into the average size distribution of synthesized NPs, the average size of particles was observed to be around 10–30 nm (Fig. 4A–E). The data obtained from EDX weight percent was used to calculate the yield of NPs formation with the help of online software [24]. In this study, we observed around 91.66 % of NPs formation from 1 mM Ag salt solution.

### 3.3 Antibacterial activity

The disc diffusion method preliminarily identified the synthesized NPs' antimicrobial potential. The antimicrobial activity was analyzed against MDR bacterial strains, namely *S. aureus*, *E. faecalis*, *E. coli*, and *K. pneumoniae*. All the MDR bacterial strains show a significant zone of inhibition. The Gram-positive bacterial strains show a zone of inhibition in the range of 18–20 mm, while for Gram-negative, it is 18–19 mm. The zone of inhibition was compared with commercially available antibiotics, namely Ampicillin and Vancomycin. Table 2 gives insight into the zone size of all the bacterial strains compared with the positive control.

**Table 2:** Zone of inhibition (mm) of AgNPs against MDR bacterial strains (±SD 3 replicates tested) Positive control for Gram-positive bacteria Vancomycin and Gram-negative bacteria Ampicillin.

Bacterial strain	Zone of inhibition (mm)	
	AgNPs	Control Positive
*S. aureus*	18.36 ± 1.51	20.66 ± 0.57
*E. faecalis*	20 ± 1	22.93 ± 0.305
*E. coli*	19.133 ± 0.808	24.83 ± 0.28
*K. pneumoniae*	18.33 ± 1.154	25.93 ± 0.11

### 3.4 Evaluation of antimicrobial activity by MIC

**Fig. 5:**
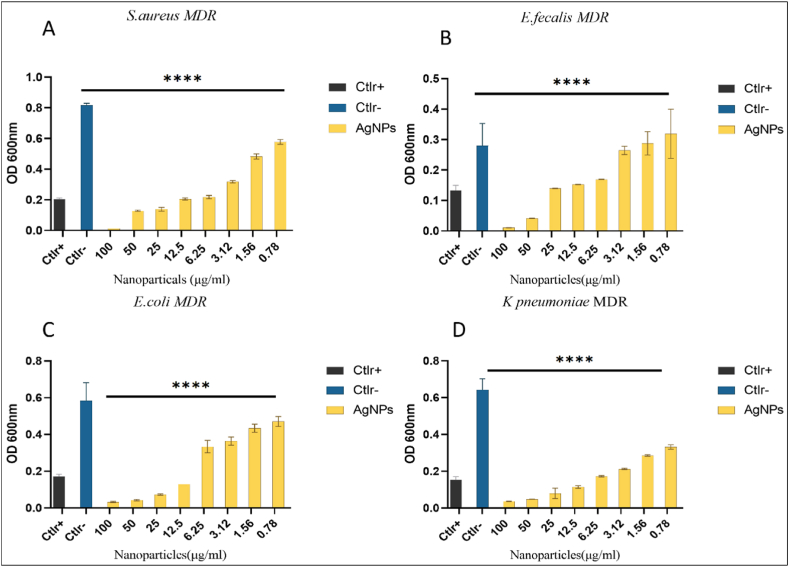
Evaluation of AgNPs by MIC against MDR bacterial pathogens. (A–B) Indicates analysis of MIC against Gram-positive bacterial strains *S. aureus* and *E. faecalis*. While (C–D) Indicates analysis of Gram-negative bacterial strains *E. coli* and *K. pneumoniae*. A positive control is indicated as Ctlr+. This study used commercially available antibiotics Vancomycin and Ampicillin against Gram-positive and negative bacterial strains. At the same time, the control negative is marked as Ctlr-i.e., untreated bacteria. Data relative to the control negative represent the mean ± standard deviation (SD). Significant differences among the mean (****p < 0.05).

Further, we performed a microdilution assay using the broth dilution method to evaluate the MIC. As a part of the systemic model, different Gram-positive and Gram-negative MDR bacterial strains, such as *S. aureus, E. faecalis, E. coli, and K. pneumoniae*. The synthesized AgNPs showed potent antimicrobial activity till the lowest concentration (3.12–6.25 μg/ml). In this study, the concentration of AgNPs ranges from 0.78 to 100 μg/ml. To carry out the MIC study, bacterial suspension of 1 × 10^−6^ CFU/ml was placed in 96 well plates. In each well, 50 μl suspension was added, followed by 20 μl of NPs ranging from 100 to 0.78 μg/ml. The plates were further incubated for 24 h at 37 °C. After 24 h of incubation, turbidity was observed in 96 well plates. The well with the least concentration showing absences of turbidity was considered the MIC value. For Gram-positive bacteria, the MIC values were around (3.12 μg/ml), while for Gram-negative, it was between (1.56–6.25 μg/ml). The mode of action of AgNPs is not yet cleared. We hypothesize that NPs attach to the bacterial cell wall, penetrate through the cell wall into a bacterial cell, and cause alteration of different metabolic pathways. It may also disrupt the DNA replication and protein synthesis process. Also, due to oxidative stress, ROS generation takes place. The results in Fig. 5 suggest that the *K. pneumoniae* are more susceptible to synthesized NPs than other bacteria mentioned. The various studies hypothesized that the antimicrobial effect of NPs is also affected by bacteria's physiological properties. Gram-positive bacteria have thick peptidoglycan layers, due to which NPs are trapped less in the cell wall. In gram-negative bacteria, NPs are more susceptible due to the thin peptidoglycan layer. It also suggests that the effectivity of antibacterial potential depends on the uptake of NPs by gram-positive and gram-negative bacteria [30]. However, the exact mechanism of action of these synthesized NPs is unknown. In the future, we will study the mechanism differences between Gram-positive and Gram-negative bacteria (Fig. 5A–D). indicates a graphical representation of NPs activity against all four MDR bacterial strains at varying concentrations. While Table 3 explains MIC values for MDR strains respectively.

**Table 3:** The minimum inhibitory concentration of monometallic NPs against all MDR bacterial strains.

Sr.No.	Bacterial strains	MIC values (μg/ml)
1.	*S. aureus*	3.15
2.	*E. faecalis*	3.15
3.	*E. coli*	6.25
4.	*K. pneumoniae*	1.56

### 3.5 Kinetic study evaluation

Time kill assay was performed to evaluate the time-dependent bacterial killing by AgNPs/NP kinetic efficiency against MDR bacterial pathogens. A significant reduction in CFU was observed after 4hrs of NPs treatments. In the case of *S. aureus*, 2MIC showed around 80 % of CFU deduction, followed by MIC showing about a 76 % reduction in CFU count after 4 h of treatments. In the case of *E. faecalis, E. coli,* and *K. pneumoniae*, it showed a significant reduction after 4 h of incubation. The multiplication rate of MDR pathogens decreased, reaching the initial number of CFUs.

**Fig. 6:**
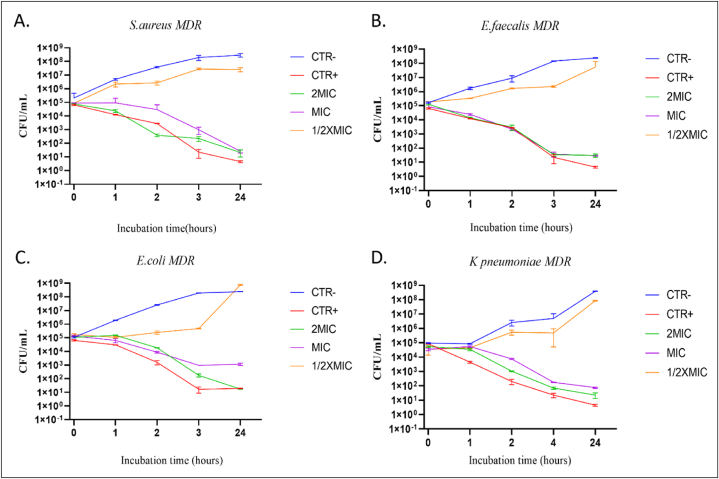
The kinetic effect of AgNPs against different MDR bacteria (A–B) Indicates Gram-positive bacteria, namely *S. aureus* and *E. faecalis*, showing a significant reduction in CFU after 4 h of incubation at 2MIC and MIC concentration. (C–D) Indicates Gram-negative bacteria, namely *E. coli* and *K. pneumoniae*, showing significant reduction in CFU after 4 h of incubation at 2MIC and MIC concentration. Control positive (Ctlr+) indicates bacteria treated with commercially available antibiotics. Vancomycin and Ampicillin were used as a positive control for Gram-positive and Gram-negative bacteria. The control negative (Ctlr-) indicates untreated bacteria. Reduction in bacterial colonies >3 log_10_ defined as bactericidal effect. The data represents three independent experiments means values ± standard deviation (±SD).

The NPs were effective against Gram-positive and Gram-negative bacteria by significantly reducing the viability of *K. pneumoniae*, *E. coli*, *S. aureus, and E. faecalis* at 2MIC and MIC concentrations. The NPs show bactericidal activity against selected Gram-positive and Gram-negative bacterial strains, significantly reducing CFU. The bactericidal curve can be observed in (Fig. 6A–D) for all the bacterial strains. The ½ MIC value for all the bacterial strains showed similar behavior to the positive control (Ctlr+).

### 3.6 Evaluation of cytotoxic effect

**Fig. 7:**
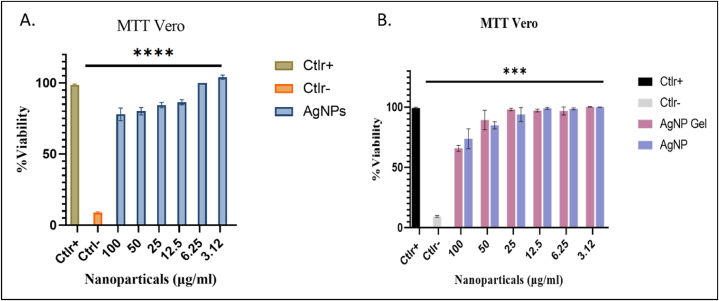
Graphical representation of cell viability NPs concentration ranges from 3.12 to 100 μg/ml, while positive control (Ctlr+) was untreated cells and control negative (Ctlr-) cells were treated with DMSO. Data is the mean of three independent experiments ****p < 0.0001 relatives to control positive (Ctlr+).

A cell viability assay (MTT assay) evaluated the NP's toxicity. In this study, the Vero cell line is used as a model cell line to examine the toxicity. After 4 h of MTT treatment, when DMSO was added to 96 well plates, viable cells turned blue, while no color change was observed for dead cells. The cells with the highest NPs concentration, around 80 % cell viability was observed (Fig. 7). The results were suggested that the synthesized AgNPs possess less toxicity as compared to other NPs [31,32].

## 4 Discussion

A novel strategy for AgNP biological synthesis using yeast extract has been developed in the present study. The aqueous (Ag+) ions reduction to metallic (Ag°) by aqueous extracellular yeast extract was discovered visually, with successful reduction indicated by a colour change from colorless to dark yellow; as the time of incubation increases, colour intensity increases. Absorption confirmed NP formation by observing the characteristic absorption maximum at 390 nm from the 300–600 nm recorded spectra. The color change attribution is due to the metallic nanostructure's outer surface electron collective oscillation in interaction with electromagnetic waves, and this phenomenon is known as surface plasmon resonances [[33], [34], [35]]. Due to Mie's scattering, colloidal AgNPs exhibit an absorption within 390–420 nm [36]. To understand the stability and size distribution of AgNPs, DLS, TEM, and Zeta potential analysis were performed.

Previous studies on *Pichia Pastoris* monometallic NPs show the size of NPs around 3.6–11.9 nm [37]. In this study, the size of NPs was observed around 48 nm with a PDI value of 0.1 by DLS. With electron microscopy it confirmed the circular morphology of NPs with size around 10–30 nm. The previous studies showed that the smaller the NPs size, the greater the antibacterial activity because smaller NPs have a more significant ability to penetrate bacterial cells. The circular-size NPs help attach and alter bacteria's cell walls and are more prone to silver release [34,35,38,39].

Along with size, zeta values are also evaluated, which gives an idea about the stability of synthesized NPs. The zeta value was observed around −22mV. The surface of the biologically synthesized NPs is covered with a negatively charged corona layer, which ensures dispersion and stability. At the same time, other studies of biological synthesis of AgNPs by yeast show a Z value around −52.18 mV [40]. Simultaneously, to confirm, the shape of AgNPs was examined with transmission electron microscopy (TEM). In this study, circular NPs with an average size of 10–30 nm are observed. It has been reported that circular-size NPs help attach and alter bacteria's cell walls [41].

After confirmation of size and shape, it is essential to understand the involvement of phytoconstituents in the synthesis process. An FTIR analysis was carried out to understand the participation of phytoconstituents. The observed functional groups at 3277 cm^−1^ - 3284 cm^−1^ may correspond to the N–H stretch of the secondary amine and the O–H stretch of alcohol. The peaks at 163 cm^−1^ - 1641 cm^−1^ and 1537 cm^−1^ -1573cm^−1^ indicate the stretching vibration of C=O and the bending of N–H in the amide bond, respectively [42]. The peak at 1398 cm^−1^ - 1388 cm^−1^ is likely due to carbohydrates’ C–H bending vibrations. Finally, 1238 cm^−1^ -1288 cm^−1^and 1047 cm^−1^ - 1068 cm^−1^ peaks correspond to the stretching of C–O and C–O–C, respectively [43]. This spectrum vibration collectively indicates that *Pichia pastoris* AgNPs are synthesized and encapsulated by phytoconstituents of yeast extract, such as different amino acids and carbohydrates [23].

The antimicrobial efficiency was evaluated of synthesized AgNPs against different clinical pathogens. The antimicrobial activity of synthesized AgNPs was evaluated against four MDR pathogens, namely *S. aureus*, *E. faecalis*, *E. coli*, and *K. pneumoniae*. Zone of clearance was observed around the disc in the disc diffusion assay. It indicated that the synthesized NPs possess antibacterial potential by inhibiting bacterial growth. A previous study by Pallavi S.S. on S. hirsutus (SNPGA-8) AgNPs shows antibacterial activity against *S. aureus*, *Pseudomonas aeruginosa*, *E. faecalis*, and *E. coli* by forming a zone of clearance around the disc [44]. Disc diffusion assay is typically performed to get a preliminary idea about the antibacterial efficiency of NPs or any antibacterial compounds. Further, to confirm the antibacterial activity, MIC analysis was required. MIC analysis provides an idea about the concentration of NPs or compounds that can inhibit bacterial growth. As indicated in Table .3, the monometallic NPs showed MIC values for Gram-positive bacteria *S. aureus* and *E. faecalis* around 3.12μg/mL. While in the case of Gram-negative bacteria *E. coli* and *K. pneumoniae*, it was observed in the range of 6.25–1.56 μg/mL, respectively. Towards *E. coli* less susceptibility was observed. This might be due to the NP's charge being neutralized by the LPS of bacteria. Hence it makes *E. coli* less susceptible to NPs [44].

The kinetic study was performed against MDR bacterial pathogens. The NPs possess bactericidal activity against selected Gram-positive and Gram-negative bacteria, and the bacterial CFU reduction was (90 %) ≥ 3 log units. The AgNPs show an efficient decrease of CFU count up to 76 % for *S. aureus* after 4 h of incubation and gradually decreased more after 24hr of incubation, reaching up to 99 % bacterial death. In this study, another Gram-positive bacteria, *E. faecalis* showed the up to (99 %) bacterial CFU reduction after 24 h at MIC concentration. Meanwhile, for 2MIC concentrations, after 4 h of treatment, 99 % of bacterial viability was reduced. The ½ MIC concentration showed quite similar behavior with the control negative (Ctlr-) in the case of both Gram-positive bacteria. While in the case of *E. coli* and *K. pneumoniae*, significant bacterial reduction was observed after 24 h of treatment for MIC values. At 2MIC values, it showed a 90 % reduction after the 4 h of NP treatment. In contrast, the ½ MIC values showed similar behavior to the control negative.

This study confirmed the bactericidal efficiency of AgNPs against all tested MDR pathogens. All the antibacterial analyses indicate that synthesized AgNPs can kill bacteria at their highest concentration, 10^−6^. In comparison, a lower concentration of NPs proves that the biologically synthesized NPs showed outstanding results. The exact mechanism of action of AgNPs needs to be explored more. Based on previous research, we hypothesized that the bactericidal activity of synthesized AgNPs is due to the electrostatic attraction and affinity towards sulfur protein. Ag^+^ ions attach to the bacterial cell wall and cytoplasm [45]. They resulted in a significant enhancement in the permeability of the cell wall, which leads to bacterial disruption or cytoplasmic leakage. Once the Ag^+^ ions are uptaken by cells, it deactivate the respiratory enzymes, leading to ROS production and interference in ATP release [46].

ROS generation could also play a vital role in aggravating the disruption of cellular membranes, and it alters the DNA. When it interacts with Ag^+^ ions, it affects DNA replication and cell propagation, and Ag ions can denature cytoplasmic ribosomal components, ultimately leading to a hindrance in protein synthesis [47].

Studies have reported that apart from releasing Ag^+^ ions, AgNPs are efficient in eradicating bacterial pathogens. AgNPs may trigger denaturation of the cell membrane, and due to the smaller size, the NPs can easily penetrate through the cell wall and causes modification in cell membrane arrangements. It may also cause organelles to rupture due to the cell denaturation of the cell membrane and may also cause cell lysis [48].

A bactericidal effect depends on the NP's shape, size, charge, and bacterial class. Despite having vigorous antimicrobial activity, nanomaterials to be utilized to prevent and treat infectious diseases should be harmless to host cells. Thus, to confirm the biocompatibility of synthesized NPs from yeast extract, a cytotoxicity assay was performed by using MTT(3-(4,5-dimethyl thiazol-2yl)-2,5-diphenyl tetrazolium bromide) against Vero cells. The average of total viable cells was precisely calculated by the MTT assay. Different concentrations of NPs at time interval of 24 h had dose dependent cytotoxic effect against Vero cells. After the 24 h of incubation 80 % cell viability was observed at highest concentration 100 μg/mL. The results suggested that the synthesized NPs are less toxic towards mammalian cells [49,50]. Finally considering the global interest in developing “Green production” to address the issue of antimicrobial resistance. Synthesis of biological AgNPs in the presence of phytoconstituents of *Pichia Pastoris* serves as a novel alternative to the nano therapy method against the MDR resistance issue. Considering advancements in nano therapy in the future, also aim to conjugate the polymeric substances on the surface of the NPs to enhance the antimicrobial effect; their antiviral potential can also be analyzed.

## 5 Conclusion

In this study, we have successfully developed a novel method for the eco-friendly and cost-effective synthesis of silver nanoparticles (AgNPs) using an extracellular extract from *Pichia Pastoris*. Our comprehensive analysis, employing techniques such as UV–Vis spectroscopy, DLS, Zeta potential, and FTIR, elucidated the formation and stability of these nanoparticles during the synthesis process. Morphological insights were gained through Transmission Electron Microscopy (TEM) Analysis.

The results revealed that the synthesized AgNPs exhibited an average size ranging from 10 to 30 nm with spherical morphology, accompanied by a negative zeta potential of −22 mV. This unique combination of small nanoparticle size and negative zeta potential contributed to their remarkable antimicrobial properties. Notably, the antimicrobial effect was evident even at low concentrations of AgNPs, demonstrating efficacy against both Gram-positive and Gram-negative bacterial strains. Further, FTIR indicated the presence of diverse phytoconstituents, plays a crucial role in stabilizing and capping the surface of the nanoparticles. This phenomenon likely contributed to the observed high cell viability, around 80 %, even at the highest concentration of nanoparticles. The advantages of our synthesized AgNPs are manifold. Not only do they boast a low production cost, but they also exhibit minimal toxicity while retaining potent antimicrobial efficacy. These findings hold great promise for various applications, particularly in the realm of nanomedicine. These studies also pave the way for following scientific research on the variations in the mode of action of AgNPs against both Gram-positive and Gram-negative bacteria.

## Funding

This research was funded by 10.13039/501100009708Novo Nordisk Foundation grant NNF20CC0035580 to I.M. and Vetenskapsrådet (2020–04096) to S.P.

## Ethical approval

Not required.

## Data availability statement

The original contributions presented in the study are included in the article; further inquiries may be directed to the corresponding author.

## Additional information

No additional information is available for this paper.

## Tested microorganisms

From the “Vanvitelli” University Hospital based in Naples, multidrug-resistant (MDR) clinical isolates were collected. Bacterial identification was carried out via MALDI-TOF MS (Bruker Daltonics, Bremen, Germany) and BD Phoenix system (Becton Dickinson, USA). Antibiotic resistance patterns were evaluated. The ATCC and other bacterial strain resistance patterns are reported in Table 1.

## CRediT authorship contribution statement

**Pragati Rajendra More:** Writing – original draft, Validation, Methodology, Investigation, Formal analysis, Data curation, Conceptualization. **Surbhi Shinde:** Writing – original draft, Investigation, Formal analysis, Data curation. **Zhejiang Cao:** Writing – original draft, Investigation, Formal analysis, Data curation. **Jian Zhang:** Investigation, Formal analysis. **Santosh Pandit:** Writing – review & editing, Writing – original draft, Visualization, Supervision. **Anna De Filippis:** Writing – review & editing, Visualization, Supervision. **Ivan Mijakovic:** Writing – review & editing, Supervision, Resources, Funding acquisition, Conceptualization. **Massimiliano Galdiero:** Writing – review & editing, Supervision, Resources, Conceptualization.

## Declaration of competing interest

The authors declare that they have no known competing financial interests or personal relationships that could have appeared to influence the work reported in this paper.
